# Simulation and Analysis of Urban Production–Living–Ecological Space Evolution Based on a Macro–Micro Joint Decision Model

**DOI:** 10.3390/ijerph18189832

**Published:** 2021-09-18

**Authors:** Yuanyuan Tao, Qianxin Wang, Yan Zou

**Affiliations:** 1 School of Environment and Spatial Informatics, China University of Mining and Technology, Xuzhou 221116, China; tyy@cumt.edu.cn (Y.T.); zouyan@bucea.edu.cn (Y.Z.); 2 School of Humanity and Law, Beijing University of Civil Engineering and Architecture, Beijing 102616, China

**Keywords:** urban space evolution simulation, cellular automata, multi-agent system, leading factors analysis, urban sustainable development

## Abstract

The precise simulation of urban space evolution and grasping of the leading factors are the most important basis for urban space planning. However, the simulation ability of current models is lacking when it comes to complicated/unpredictable urban space changes, resulting in flawed government decision-making and wasting of urban resources. In this study, a macro–micro joint decision model was proposed to improve the ability of urban space evolution simulation. The simulation objects were unified into production, living and ecological space to realize “multiple planning in one”. For validation of the proposed model and method, remote sensing images, geographic information and socio-economic data of Xuzhou, China from 2000 to 2020 were collected and tested. The results showed that the simulation precision of the cellular automata (CA) model was about 87% (Kappa coefficient), which improved to 89% if using a CA and multi-agent system (MAS) joint model. The simulation precision could be better than 92% using the prosed model. The result of factor weight determination indicated that the micro factors affected the evolution of production and living space more than the macro factors, while the macro factors had more influence on the evolution of ecological space than the micro factors. Therefore, active policies should be formulated to strengthen the ideological guidance towards micro individuals (e.g., a resident, farmer, or entrepreneur), and avoid disordered development of living and production space. In addition, ecological space planning should closely link with the local environment and natural conditions, to improve urban ecological carrying capacity and realize urban sustainable development.

## 1. Introduction

Land use/cover change (LUCC) has been generally considered a main driving force of global ecosystem and climate change [1]. Urbanization is the most typical form of LUCC, and has a significant impact on biological diversity and ecosystem services [2]. Nowadays, about 55% of the world’s population lives in cities with this rate expected to reach 68% by 2050 [3]. Therefore, the study of urban LUCC is of great importance to understand and grasp global LUCC. The process of urbanization influences the flow of material, energy, and information, and affects the structure and function of ecosystems [4]. Therefore, on the one hand, the socio-economic level may be significantly improved; on the other hand, it leads to loss of farmland, fragmentation of habitats, and increases in the heat island effect [5,6,7]. However, these problems can be alleviated through reasonable urban space planning and efficient utilization of urban resources [8]. Therefore, urban space planning is considered an effective tool/means to improve urban sustainable development.

The precise simulation of urban space evolution and analysis of the leading factors are the most important bases and essential prerequisites for urban space planning. Therefore, many studies of urban space evolution simulation and leading factors analysis have been carried out, and some typical simulation models have been constructed, such as the econometric statistics model (ES) [9], the system dynamics model (SD) [10], the cellular automata model (CA) [11], the multi-agent system model (MAS) [12], etc. Each of these models has its own advantages and disadvantages. In ES models, mathematical statistics methods are used to simulate the variations in scale of urban space, such as the logistic regression model [13], Kuznets curve model [14], and panel econometric model [15]. These ES models are easy to construct and use, but they cannot simulate the dynamic process and variation in urban spatial distribution. In SD models, the process of urban space evolution is expressed by simulating the interactions of urban elements, e.g., MEPLAN model [16], Dortmund model [17] and LILT model [18]. However, these SD models also cannot describe variation in urban spatial distribution. The ES and SD models are considered “top-down” models.

To improve the ability to simulate urban spatial variation, the CA model was first introduced by Chapin and Weiss in 1968 [19]. From the late 1990s to the early 21st century, the CA model entered a high-speed development period, and saw widespread use in urban space planning [20,21]. However, it cannot well represent macro-scale political, economic and cultural driving forces that influence urban spatial variations [22]. Therefore, some improved methods have been proposed, for example taking the macro factors as constraint conditions in the CA model [23]. With the development of artificial intelligence (AI) technology, the study and application of the MAS model became popular over the past two decades. MAS defines a set of agents living in a common environment, with all agents coming to a joint decision on urban space use type within this system. The MAS model can provide a more powerful tool for simulating the multi-level decision-making processing in urban space evolution than the other existing methods [24,25]. However, the MAS model has to be used in conjunction with CA for considering the effects of neighborhood space use type on urban space evolution [26]. Both CA and MAS are considered “bottom-up” models. Table 1 shows the comparison of the characteristics of the four kinds of models.

From Table 1, it is apparent that the simulation ability of the MAS model is stronger than that of other models. However, there are still some defects in the current MAS model. For example, the interaction between macro and micro factors is rarely considered, despite having a significant effect on the simulation precision of the model. Macro factors (e.g., the natural condition, government policy) often restrict the decision-making behaviors of micro factors (a resident, farmer, entrepreneur or environmentalist), particularly in developing countries [27]. The choice preferences of micro factors may also have an important influence on the decision-making behaviors of macro factors [28]. If the interaction and mutual influence between macro and micro factors are neglected, the simulation precision of the model will inevitably decrease. Moreover, the simulation objects are usually unit plots of different types of land use (e.g., urban construction land, cultivated land, woodland, grassland, water bodies, etc.) in current models. If the types of land use are different from the categories of urban planning (e.g., traffic planning, garden planning, land planning) for the same unit plot at the same time, it will lead to land use conflicts.

To solve these problems, a macro–micro joint decision model is proposed in this study for improving the simulation ability of urban space evolution. In this model, all simulation objects are unified into production, living and ecological space for realizing “multiple planning in one”. Compared with current models, the proposed model has two main characteristics. One is that the interaction and mutual influence between the macro and micro factors are fully considered; the other is that “Production–Living–Ecological” (PLE) space is taken as the simulation object, replacing exclusive types of land use. For validation of the proposed model, remote sensing images, geographic information and socio-economic data from Xuzhou, China between 2000–2020 were collected and tested. The results show that the simulation precision of CA model was 87.14% (Kappa coefficient), with an increase to 89.63% when using CA + MAS model. An additional 2.68% improvement of simulation precision was achieved by using the CA + MAS + Correlation model. Moreover, the result of factor weight determination indicated that the micro factors affected the evolution of production and living space more than the macro factors. However, the macro factors had more influence on the evolution of ecological space evolution than the micro factors. Therefore, we should pay more attention to the micro factors to realize the orderly development of living and production spaces. For example, some active policies should be formulated to strengthen the ideological guidance for micro individuals (e.g., residents, farmers, entrepreneurs), helping them establish scientific views of urban space utilization. Meanwhile, macro factors should be paid more attention to ensure the sustainable development of ecological space. For example, ecological space planning should closely link with the local environment and natural conditions to improve the urban ecological carrying capacity.

## 2. Study Area and Data Sources

### 2.1. Study Area

**Figure 1:**
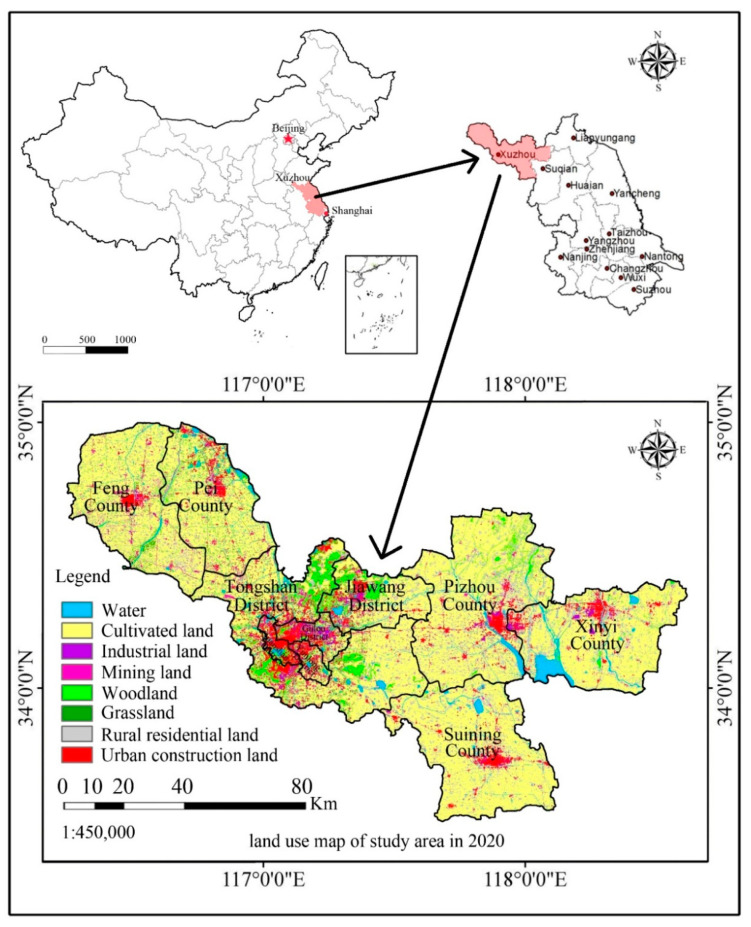
The geographical location and administrative divisions of Xuzhou in China.

To analyze the leading factors and construct a precise simulation model of urban space evolution, Xuzhou, an eastern city of China, was selected as the study area. It is located in the northwest of Jiangsu Province, China (between 33°43′–34°58′ N and 116°22′–118°40′ E). It includes five districts, three counties and two county-level cities, with a total area of 11,765 km^2^. Figure 1 shows the geographic location and administrative divisions of Xuzhou. The majority of the Xuzhou region consists of plains, which account for 90% of the total area. This area has a temperate continental monsoon climate and receives 44–54% possible sunshine. The annual average temperature is 14 °C, and the annual average rainfall is 900 mm. In addition, Xuzhou is rich in mineral resources and well placed for easy access to the other Chinese cities. Therefore, it is an important coal production base and a transportation hub in China [29]. In the past two decades (2000–2019), the population of Xuzhou increased from 8,964,400 to 10,417,300, and the Gross National Product (GDP) improved from 61.630 billion CNY to 715.135 billion CNY; the per capita green area increased from 10.5 m^2^ to 15.4 m^2^, and the urbanization rate increased from 25.8% to 66.7% [30].

From the above statistics, it is evident that the socio-economic level and living standards of Xuzhou have improved significantly in the past two decades. The types of land use/coverage have changed significantly in Xuzhou, which can provide a good basic dataset to investigate the process of urban space evolution and leading factors. However, it should be noted that the level of economic development is still low and the cost of economic development is high in Xuzhou compared with other cities in Jiangsu Province. In 2018, the per capita GDP of Xuzhou was 76,915 CNY, which was 66.88% of the average level of Jiangsu Province, but the comprehensive energy consumption of Xuzhou was 0.37 tons (consumed standard coal for obtaining 10,000 CNY of industrial output), which was 3.12 times the average level of Jiangsu Province [31]. Therefore, more energy needs to be consumed in order to realize the same amount of economic growth in Xuzhou as in other cities. Therefore, it is important and urgent to strengthen the urban space planning and optimize the industrial structure as soon as possible, to realize the sustainable development of social economy and the ecological environment in Xuzhou.

### 2.2. Data Sources

In this study, three kinds of data were collected and used: the first was remote sensing (RS) images of Xuzhou from 2000 to 2020, which provided the basic data for Production-Living-Ecological (PLE) space recognition; the second was geographic information system (GIS) data, which provided vector files of the administrative divisions, traffic networks and distribution of public facilities in Xuzhou, such as schools, hospitals, and shopping malls, as downloaded from a geographic national conditions monitoring cloud platform; the third was socio-economic statistical data from the literature and statistical yearbooks from 2000 to 2020, including population, industrial economy, natural resources, etc. Details on the three types of data are listed in Table 2.

It should be noted that the RS images needed to be chosen and processed carefully in order to obtain the precise urban space evolution information. Therefore, RS image data was collected during summer (from mid-July to mid-August), because identifying RS images of vegetation in the growth season peak is easier than during other seasons [31]. Environment for Visualizing Images (ENVI) software (Research System Comp., Boulder, CO, USA) was used for data processing. The main procedures included radiative correction, atmospheric correction, geometric correction, contrast stretching, graphics clipping, etc. Finally, the land was classified into urban construction land, rural residential land, cultivated land, woodland, grassland, water area, industrial land, and mining land by the supervised classification and visual interpreted method. To improve the accuracy of land use classification, convolutional neural network (CNN) technology was adopted [32]. Through land function evaluation, the above eight types of land use could be amalgamated into production, living and ecological space [33]. The recognition precision of PLE space was better than 96% (Kappa coefficient) based on the RS image data. Therefore, it could meet the requirements of PLE space evolution simulation and leading factor analysis.

## 3. Research Methods

### 3.1. Production-Living-Ecological Space Evolution Simulation Model Based on Macro-Micro Joint Decision

**Figure 2:**
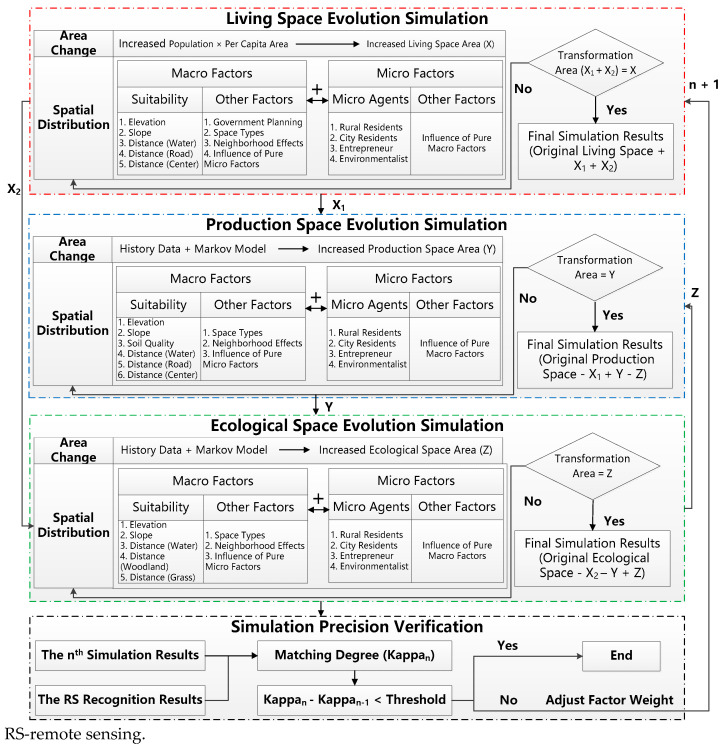
The data processing flow for simulating Production–Living–Ecological space evolution based on the macro and micro factor joint decision model.

According to Section 1, many models have been constructed to simulate urban space evolution, and the simulation ability of CA + MAS is the strongest among the current models. However, there are still some defects in the CA + MAS model; for example, the correlations between macro and micro factors are neglected. To improve the performance of the CA + MAS model, a macro–micro joint decision model is proposed in this study. Compared with the current CA + MAS model, the proposed model has two main characteristics. One is that the interaction between and mutual influence of the macro and micro factors are considered to improve the simulation precision over the CA + MAS model; the other is that PLE space is taken as the planning object to facilitate the unified implementation of multiple planning. The specific steps of the proposed model are as follows: (i) Areas of increased living space are obtained by predicting the growth of the urban population in the future. (ii) Locations of increased living space are determined by the CA + MAS model. It should be noted that the probability of the *i*^th^ unit space to be transformed into living space is calculated by the joint decision of macro and micro factors; the highest-probability unit space is transformed into living space. (iii) After completing the transformation of the *i*^th^ unit space, the model checks if the total of transformed area is equal to the area of increased living space. If not, the above work is done iteratively until the total transformed area is equal to the area of increased living space. (iv) After completing the simulation of living space evolution, production and ecological space evolution are simulated using the same method. However, the areas of increased production and ecological space are instead determined by historical data and a Markov model. (v) After completing the *n*^th^ simulation of PLE space evolution, the simulation precision is calculated (Kappa_n_) by comparing with the results of RS image recognition. If the value of (Kappa_n_ − Kappa_n−_) is smaller than the threshold, the solution has converged and the *n*^th^ simulation result is outputted. If not, the weights of macro and micro factors aree adjusted and the above steps are done iteratively. The method of factor weight adjustment is introduced below in Section 3.2. Figure 2 shows the data processing flow of PLE space evolution simulation based on the macro and micro factor joint decision model.

#### 3.1.1. Simulation Method of Living Space Evolution

According to Figure 2, the areas of increased living space should be determined first in order to simulate the evolution of urban space. Two methods are often used to realize this. One is based on the historical data (areas of increased living space in previous years) and a Markov model. The other is based on predicting the growth of the urban population and the per capita area of living space in the region. Existing studies indicate that the estimation precision of areas of increased living space from the latter method is better than that from the former method, if the urban population is predicted accurately [34]. Therefore, the latter method is adopted in this study and the calculation formula is:

(1) $$A^{k}=P^{k}\times S$$

*A^k^* and *P^k^* are the areas of increased living space and urban population in the *k^th^* year, respectively. *S* is the per capita area of living space, which refers to the historical data of this city or its urban planning and design standards. In China, the per capita area of living space is divided into seven types, according to the urban population size and climatic conditions. The per capita area of living space ranges from 65 m^2^ to 115 m^2^ based on the different type of city. Xuzhou belongs to the second type of city, where the per capita area of living space is 110 m^2^.

After obtaining the area of increased living space, one of most important problems is determining the locations of these increases. In this model, locations of increased living space are determined by the joint decision of macro and micro factors. The greater the transformation probability of unit space, the higher its priority for transformation into living space. The calculation formula is:

(2) $$F_{i,j}^{k}=W^{macro}F_{i,j}^{’k}+W^{micro}F_{i,j}^{’’’k}$$

*F^k^_i,j_* is the probability of unit space (*i, j*) to be transformed into living space in the *k^th^* year. For convenience, probability is replaced with a score, where 0–100 scores denote 0%–100%. *F^’k^_i,j_* and *F^’’’k^_i,j_* are the scores from the macro and micro factors, respectively. *W^macro^* and *W^micro^* are the weights of macro and micro factors, respectively, where *W^macro^* + *W^micro^* = 1. The initial values of *W^macro^* and *W^micro^* can be determined by statistical analysis. The specific implementation steps are introduced below in Section 3.2. Final weights of the macro and micro factors are adjusted by comparing the simulation results with RS image recognition results. The scores from macro factors include two components: suitability and all other factors. The calculation formula is:

(3) $$F_{i,j}^{’k}=W^{E}E_{i,j}+W^{Q}Q_{i,j}^{k}$$

*E_i,j_* and *Q^k^_i,j_* are the scores of unit space (*i*, *j*) from the suitability evaluation and the other factors, respectively. *W^E^* and *W^Q^* are their weights and *W^macro^* + *W^micro^* = 1. The calculation formula is:

(4) $$E_{i,j}=\sum_{m=1}^{n}W^{m}F_{i,j}^{m}$$

*F^m^_i,j_* and *W^m^* are the score and weight of the *m*^th^ factor, respectively. In this model, the factors of suitability evaluation include two parts: one is denoted the natural factor (e.g., elevation, slope); the other is denoted the location factor (e.g., the distances to water, main road and city center). These can be obtained with ArcGIS (ESRI, Redlands, CA, USA). The specific marking standards are as follows: the smaller the difference between the elevation of the unit space (*i*, *j*) and the average elevation of the region, the higher the likelihood of its transformation into living space. As the height difference increases from 0 to 10 km, the suitability score decreases from 100 to 0. The steeper the slope, the lower the score; as the slope increases from 0 to 90 degrees, the score decreases from 100 to 0. Likewise, the shorter the distance to a water area (0–5 km), the lower the score (0–100); the shorter the distance to a main road (0–5 km), the higher the score (100–0); and the shorter the distance to the city center (0–50 km), the higher the score (100–0). The calculation formula of scores from the other factors is:

(5) $$Q_{i,j}^{k}=W^{1}F_{g}+W^{2}F_{l}^{k-1}+W^{3}F_{N}(\sum_{m_{2}=1}^{n_{2}}N_{i,j}^{m_{2}})+W^{4}F_{q}(\sum_{m_{3}=1}^{n_{3}}w^{m_{3}}q_{i,j}^{m_{3}})$$

*F_g_*, $F_{l}^{k-1}$, *F_n_*, *F_q_* and *W*^1^, *W*^2^, *W*^3^, *W*^4^ are the scores and weights from government planning, land type, neighborhood influence, and influence of micro factors, respectively. *W*^1^ + *W*^2^ + *W*^3^ + *W*^4^ = 1. The specific marking standards are as follows: If the unit space belongs to the first, second, or third class of urban construction land in government planning, the score of *F_g_* is 100, 80, or 50 respectively. If the probability of *l*^th^ type of land to be transformed into urban construction land is between 0 and 1 in the (*k*−1)^th^ year, the score of $F_{l}^{k-1}$ is correspondingly between 0 and 100. If the number of living spaces permitted is between 0 and 8 (in a 3 × 3 neighborhood), the score of *F_n_* is correspondingly between 0 and 100. The score of *F_q_* is a weighted average of the scores of $q_{i,j}^{m_{3}}$, where *m*^3^ = 4 (rural resident, city resident, entrepreneur and environmentalist), and *w*^1^ + *w*^2^ + *w*^3^ + *w*^4^ = 1. The calculation formula of $F_{i,j}^{’’’k}$ in Equation (2) and the marking standards of $q_{i,j}^{m_{3}}$ are as follows:

(6) $$F_{i,j}^{’’’k}=W^{F^{\prime \prime }}F_{i,j}^{’’k}+W^{F^{\prime }}F_{i,j}^{’k}$$

$F_{i,j}^{’’k}$ denotes the scores of the pure micro factors, which are calculated by Equation (7). $F_{i,j}^{’k}$ is the scores from the macro factors in Equation (3). $W^{F^{\prime \prime }}$ and $W^{F^{\prime }}$ are their weights, and $W^{F^{\prime \prime }}$ + $W^{F^{\prime }}$ =1.

(7) $$F_{i,j}^{’’k}=\overset{n_{3}}{\underset{m_{3}=1}{\sum }}w^{m_{3}}q_{i,j}^{m_{3}}$$

The meanings of the symbols in Equation (7) are the same as those in Equation (5). The marking standards of $q_{i,j}^{m_{3}}$ can be introduced in detail: The shorter the distance to cultivated land (0–10 km), the lower the score (0–100) from the rural resident agent. The shorter the distance to public facilities (e.g., hospital, school, mall, 0–10 km), the higher the score (100–0) from the city resident agent. The higher the housing price per unit of space (0–20,000 CNY), the higher the score (0–100) from the enterprise agent. The shorter the distance to ecological space (0–10 km), the lower the score (0–100) from the environmentalist agent. Through Equations (1)–(7), the scores of all unit spaces transformed into living space can be obtained. The highest scoring unit space is then transformed into living space. If the accumulated transformed area is smaller than the area of increased living space (*X*), the above work is done iteratively.

#### 3.1.2. Simulation Method of Production and Ecological Space Evolution

After completing the simulation of living space evolution, production and ecological space evolution is simulated by the above method. Similarly, the areas of increased production and ecological space need to be determined first. In general, they are determined by historical data and a Markov model. If the areas of increased production and ecological space are *Y* and *Z*, and the areas of production and ecological space transformed into living space are *X*_1_ and *X*_2_ (*X*_1_ + *X*_2_ = *X*), the following equation can be employed:

(8) $$\left(S_{L}+X_{1}+X_{2}\right)+\left(S_{P}-X_{1}-Z+Y\right)+\left(S_{E}-X_{2}+Z-Y\right)=S_{L}+S_{P}+S_{E}$$

*S_L_*, *S_P_* and *S_E_* are the areas of original living, production and ecological space, respectively. Equation (8) requires that the total area before and after transformation be equal, and the method of determining the locations of increased production and ecological space is the same as that for increased living space. However, their factors and marking standards are different. In the simulation of production space evolution, the macro factors include the natural factor (elevation, slope, soil quality), location factor (distance to water area, main road, city center), and the other factors. The marking standards are: the larger the height difference between the elevation of unit space (*i*, *j*) and the average elevation of the region (0–10 km), the lower the score for its transformation into production space (0–100); the steeper the slope (0–90 degrees), the lower the score (0–100); the higher the soil quality (level 1–10), the lower the score (100–0); the shorter the distance to a water area (0–5 km), the higher the score (100–0); the shorter the distance to a main road (0–5 km), the higher the score (100–0); and the shorter the distance to the city center (0–50 km), the higher the score (100–0). The other factors include the land type, the neighborhood and the influence of micro factors. The marking standards of land type and neighborhood influence are similar to those of increased living space. However, the influence rules of micro factors are different to those of increased living space.

In the simulation of production space evolution, the shorter the distance to cultivated land (0–10 km), the higher the score (100–0) from the rural resident agent. The shorter the distance to living space (0–10 km), the lower the score (0–100) from the city resident agent. The higher the cost of land (0–10,000 CNY), the lower the score (100–0) from the enterprise agent. The shorter the distance to ecological space (0–10 km), the lower the score (0–100) from the environmentalist agent. Following the above rules, the final score of each unit space can be obtained from the weighted average of the scores of all factors. The highest-scoring unit space is transformed into production space. If the accumulated transformed area is smaller than the area of increased production space (*Y*), the above work is done iteratively.

In the simulation of ecological space evolution, the macro factors also include the natural factor (elevation, slope), location factor (distance to water area, woodland, grassland), and the other factors. The marking standards are: the larger the height difference between the elevation of unit space (*i*, *j*) and the average elevation of the region (0–10 km), the higher the score for its transformation into ecological space (100–0); the steeper the slope (0–90 degrees), the higher the score (100–0); the shorter the distance to a water area (0–5 km), the higher the score (100–0); the shorter the distance to woodland (0–5 km), the higher the score (100–0); and the shorter the distance to grassland (0–5 km), the higher the score (100–0). The other factors include the land type, the neighborhood and the influence of micro factors. The marking standards of land type and neighborhood influence are similar to those of increased living space. However, the influence rules of micro factors are different.

The shorter the distance to cultivated land (0–10 km), the lower the score (0–100) from the rural resident agent for the simulation of ecological space evolution. The shorter the distance to living space (0–10 km), the higher the score (100–0) from the city resident agent. The shorter the distance to production space (0–10 km), the lower the score (0–100) from the enterprise agent. The shorter the distance to ecological space (0–10 km), the higher the score (100–0) from the environmentalist agent. The highest-scoring unit space is transformed into ecological space, and the above work is done iteratively until the accumulated transformed area is equal to the area of increased ecological space (*Z*).

### 3.2. Method of Factor Weight Determination

In general, factor weight determination includes two main steps: the first step is the initial weight determination and the second step is the final weight adjustment. The initial weights of all factors can be determined through the Delphi method (expert scoring) or through statistical analysis. Although the Delphi method is easy to use, it has strong subjectivity. Therefore, the statistical analysis method is adopted in this study. The implementation steps of this method are introduced as follows, taking living space as an example: (i) One hundred experimental units are selected as the samples and they are evenly distributed across the study region. The area of each sample unit is 3 km × 3 km, which include 10,000 grids (30 m × 30 m). (ii) Those grids are picked out which belonged to production or ecological spaces in 2000. If the number of grids is *n*, *n* equations can be formed in one sample unit; see Equation (9).

(9) $$\{\begin{array}{c} W_{1}F_{1}^{1}+\ldots W_{j}F_{j}^{1}+\ldots W_{m}F_{m}^{1}=V^{1}\\ \vdots \\ W_{1}F_{1}^{i}+\ldots W_{j}F_{j}^{i}+\ldots W_{m}F_{m}^{i}=V^{i}\\ \vdots \\ W_{1}F_{1}^{n}+\ldots W_{j}F_{j}^{n}+\ldots W_{m}F_{m}^{n}=V^{n} \end{array}$$

*W_j_* is the weight of the *j*^th^ factor, which is a known parameter; $F_{j}^{i}$ is the score of the *j*^th^ factor in the *i*^th^ grid, which can be obtained by the methods in Section 3.1; *V^i^* is the total score of the *i*^th^ grid, which can be determined based on the time order of the transformation into living space. The earlier the grid is transformed into living space (from 2001 to 2010), the higher the score (from 100 to 0), which can be obtained by comparing the results of RS image recognition in different years. For example, if the *i*^th^ grid was transformed into living space in 2001, *V^i^* is 100; if it was transformed into living space in 2002, *V^i^* is 90; if it had not been transformed into living space in 2010, *V^i^* is 0. *m* is the number of factors for living space evolution simulation, which is 16 in this study (see Table 5). *n* is the number of grids which belonged to production or ecological spaces in one sample unit in 2000. In general, *n* is larger than *m*. In this example, *n* is approximately 6000. Therefore, in order to strengthen the stability of solutions, *n* equations of one sample unit are divided into *k* groups, with each group including approximately 200 equations. The unknown parameters (factor weight *W_j_*) of each group can then be estimated by the adjustment method (e.g., least square adjustment, LSQ), and solution precision (root mean square error, RMS) can be obtained. If the RMS of this group is more than three times larger than the minimal RMS of all groups, it is treated as an outlier and the estimated results of this group are removed. Then, the weighted average values of the remaining groups are taken as the estimated results of this sample unit. (iii) Finally, the estimated results of one hundred units are analyzed by statistical methods (e.g., Shapiro–Wilk Test [35]). If the estimated result of the factor shows a normal distribution, the weighted average value of all units is taken as the initial weight of this factor. Otherwise, sample units are deleted from the samples where the estimated values obviously deviate from the mean value of all samples, and the estimated results are tested for a normal distribution again.

**Figure 3:**
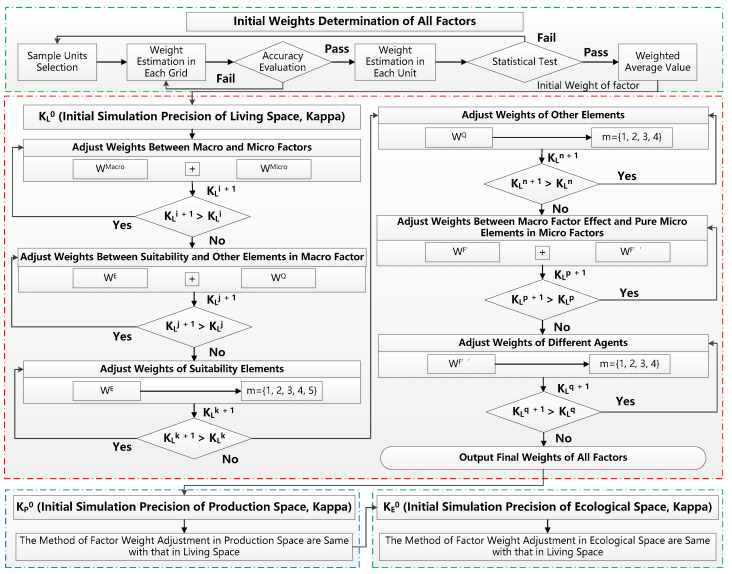
The data processing flow of factor weight determination using statistical analysis and best-fit method. The meanings of symbols are same with those in Equations (1)–(9).

After the factor initial weights are obtained, they must be further adjusted to determine the final weights, because the estimated precision and reliability of factor initial weight is strongly related to the sample selection. Therefore, they need to be adjusted by comparing the overall consistency of simulation and recognition results from the study region. In this model, the best-fit method is adopted to determine the final weights of all factors. The basic idea is that the results of simulation are always compared with those of RS image recognition, in order to test the rationality of factor weight allocation. For example, if the initial weights of macro and micro factors are 0.6 and 0.4, they will be reset to 0.59 and 0.41 on the first try. If the matching rate (Kappa) improves, they will be further set to 0.58 and 0.42 in the second try and similar attempts will be carried out until the matching rate starts to decline. Otherwise, if the matching rate in the first try is declined, the weights of macro and micro factors will be adjusted in the opposite direction (e.g., be set to 0.61 and 0.39) and similar attempts will be carried out until the matching rate starts to decline. The purpose of this method is to hunt for the optimal weight allocation of factors by a continuous adjustment. However, some rules of weight adjustment are defined to improve operating efficiency: (i) the overall weights between macro and micro factors must be adjusted first, followed by the local weights of internal elements in macro and micro factors; (ii) the lower the matching rate of space simulations, the more likely the factor weight of this type of space is adjusted first. Based on the above rules, the implementation steps of this method are as follows: (i) The matching rates of production, living and ecological spaces are calculated, based on the initial weights. (ii) The weights of macro and micro factors are adjusted first in the type of space with the lowest matching rate. If the matching rate improves, the adjustment is increased. If not, the adjustment is repeated in the opposite direction and increased until the matching rate starts to decline. (iii) Then, the weights of internal elements in the macro and micro factors are adjusted, using a method similar to that of (ii). (iv) Finally, the weights of macro factors, micro factors and their internal elements are adjusted one by one in the spaces with the second highest and highest matching rates. After the weights of all factors are determined, the effect of each factor on PLE space evolution can be analyzed by its weight. Figure 3 shows the data process flow of factor weight determination by the statistical analysis and best-fit method.

## 4. Experimental Results and Analysis

### 4.1. Simulation and Prediction Results of Production-Living-Ecological Space Evolution

**Figure 4:**
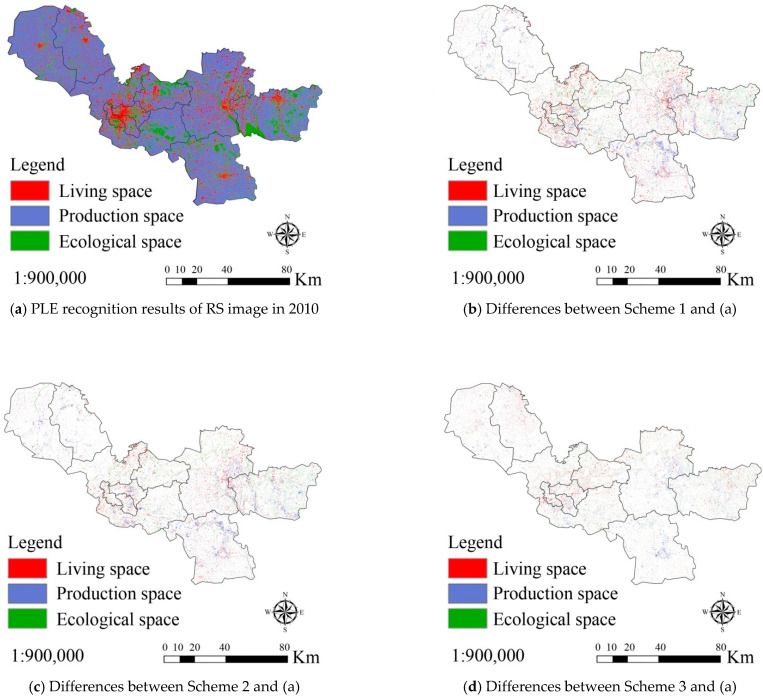
Differences between production-living-ecological (PLE) space evolution results from three experimental simulation schemes and remote sensing image recognition in Xuzhou, China in 2010. (**a**) is PLE recognition results of RS image in 2010; (**b**–**d**) are the differences between the simulation results of scheme 1, 2, 3 and the recognition results of (**a**), respectively.

For validation of the proposed model and method in Section 3, GaoFen-2 (GF) remote sensing images, geographic information and socio-economic data from Xuzhou, China between 2000 and 2020 were collected. Three experimental schemes were designed. In scheme 1, the results of PLE space recognition from RS imaging in 2000 were taken as the basic data, and used to simulate the PLE space evolution in 2010 based on the CA model. The simulation precision was obtained by comparing its results with the result of RS image recognition in 2010. In schemes 2 and 3, the basic data were the same as in scheme 1. However, the CA + MAS model and the macro–micro joint decision model (CA + MAS + Correlation) were adopted in schemes 2 and 3, respectively. Figure 4 shows the spatial distribution of patches where the simulation results of the three schemes were different from the results of RS imaging. Table 3 shows the statistical results of the simulation precisions (Kappa coefficient) of the three schemes.

From Figure 4 and Table 3, it can be seen that the simulation precision of the CA model was poor (87.14%) and that most of errors resulted from the simulation of living space evolution. The simulation precision of production space evolution was better, and that of ecological space evolution was best. In scheme 2, the simulation precision of PLE space evolution was improved to 89.63% using the CA + MAS model. The precision improvement in living space evolution simulation was the maximum. In scheme 3, the simulation precision of PLE space evolution increased to 92.31% by using the CA + MAS + Correlation model. These experimental results prove that the proposed model has a stronger ability to simulate PLE space evolution than the current models (e.g., CA and CA + MAS).

**Figure 5:**
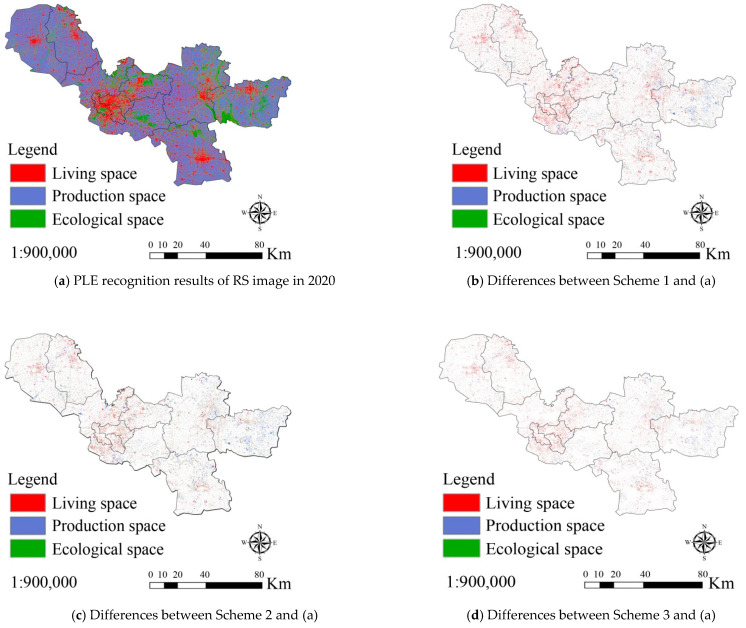
Differences between PLE space evolution predictions from three experimental schemes and that determined from remote sensing image recognition in Xuzhou, China in 2020.

To further verify the prediction ability of PLE space evolution of our proposed model, three further experimental schemes were designed. In scheme 1, the recognition result of PLE space from RS imaging in 2010 was taken as the basic data source. It was used to predict the PLE space evolution in 2020 based on the CA model, and the prediction precision was calculated by comparison with the results of RS image recognition in 2020. It should be noted that the weights of all factors remained unchanged in the prediction, and were derived from the simulation results comparing 2000 to 2010. The main reason was that the actual distribution of PLE space in 2020 could not be known in advance during the prediction. Therefore, the weights of all factors could not be adjusted over time in the simulation. This led to a decrease in prediction precision. In schemes 2 and 3, the data and method were the same as those used in scheme 1. However, the CA + MAS model and the CA + MAS + Correlation were adopted to predict PLE space evolution in 2020 in schemes 2 and 3 respectively. Figure 5 shows the spatial distribution of patches where the prediction results of three schemes were different from the recognition results from RS imaging in 2020. Table 4 is the statistical result of the prediction precisions of the three schemes.

From Figure 5 and Table 4, it can be seen that the prediction precisions of PLE space evolution were lower than their simulation precisions in the earlier schemes, as the factor weights from the prediction models were replaced with those from the simulation models. This inevitably leads to errors. However, the prediction precision of the CA + MAS + Correlation model remained better than that of the CA and CA + MAS models, and the improvement in prediction precision was larger than that in simulation precision, after considering the interactions of macro and micro factors. This proves that the proposed model had a stronger ability to predict PLE space evolution than the CA and CA + MAS models. Thus, the future distribution of urban PLE space may best be predicted by the CA + MAS + Correlation model. Policy makers and city administrators can determine the problems of urban space development in advance based on prediction results, and some positive policies can be formulated to avoid these problems and realize the sustainable development of urban PLE space.

### 4.2. Results of Factor Weight Determination

**Figure 6:**
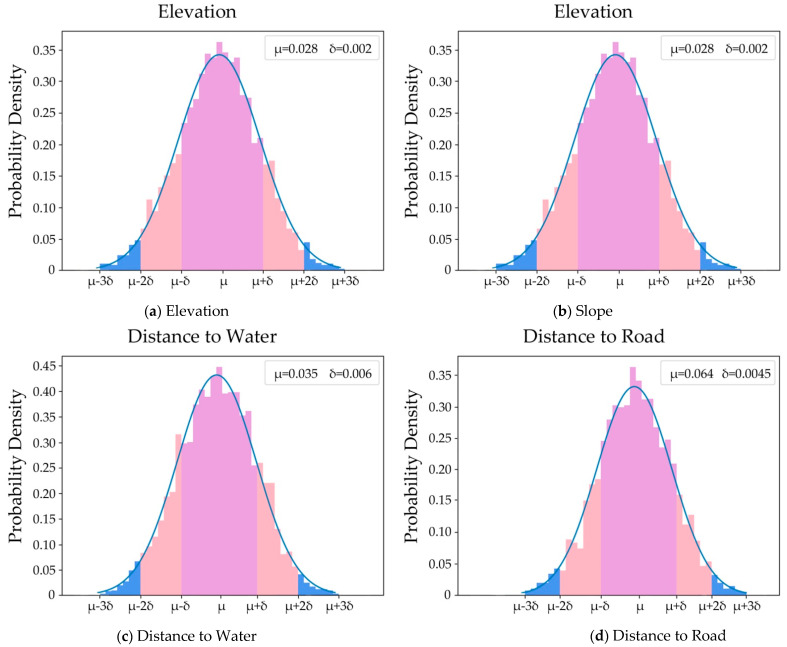
Error distributions of initial weight estimation of Elevation (**a**), Slope (**b**), Distance to Water (**c**) and Distance to Road (**d**) in living space evolution simulation.

**Figure 7:**
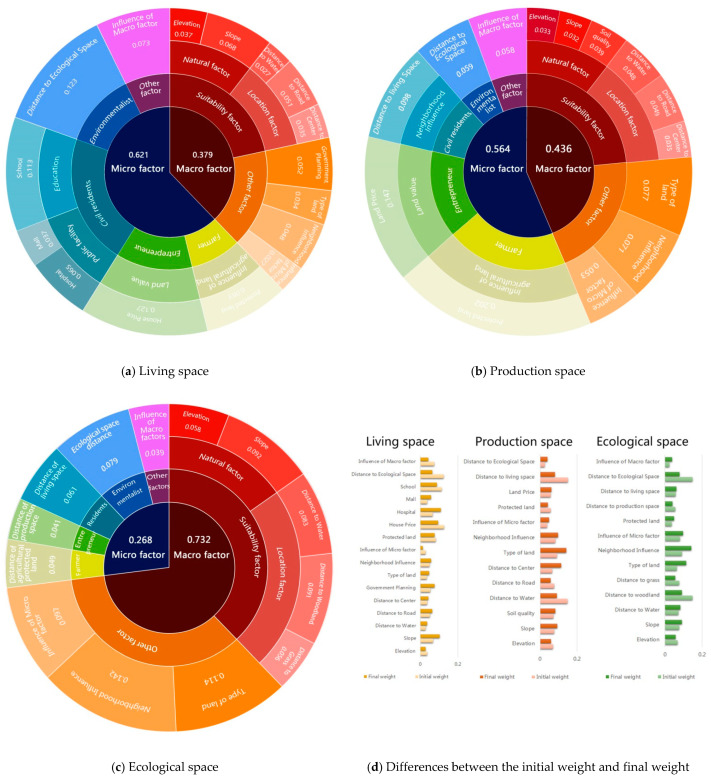
Final weight determination results of living space (**a**), production space (**b**) and ecological space (**c**) evolution simulation factors and their differences with the initial weights (**d**).

The initial weights of all factors can be obtained by statistical analysis methods and the final weights are determined by the best-fit method, as introduced in Section 3.2. The effect of each factor on PLE space evolution can be obtained based on the weight determination results. It is helpful for policy makers to grasp the leading factors and formulate scientific planning of urban PLE space development. Table 5, Table 6 and Table 7 show the initial weight determination results of factors using the CA + MAS + Correlation model to simulate the evolution of production, living and ecological space as described in Section 4.1, respectively. Figure 6 shows the error distribution of the estimated initial weights of some factors (e.g., elevation, slope, distance to water and distance to road) in the simulation of living space evolution. Figure 7 shows the final weight determination results of all factors in the simulation of production, living, and ecological space evolution and their differences with the initial weights, respectively.

From Figure 5 and Figure 6, it is known that the initial weights of all factors could be obtained by statistical analysis methods and that the estimated results were reliable. In general, the weights obeyed normal distributions, although the estimated precisions were different. Some were high (e.g., elevation and distance to water), and some were low (e.g., slope and distance to road), because the estimated precision of factor initial weight was strongly related to the sample selection. Therefore, it is a key to selecting representative and diverse samples.

From Figure 7, it can be seen that the final weights of all factors had some changes from the initial weights in Table 5, Table 6 and Table 7. The reason was that the local consistency of simulation and recognition results from the samples was exclusively considered in the initial weight determination, while the overall consistency from the study region was emphasized in the final weight determination. It is noted that the differences between the initial and final weight determination were not obvious, proving that the initial weight determination and sample selection were suitable for this study. Based on the results of factor weight determination, the effect of each factor on PLE space evolution could be obtained. The micro factors affected the evolution of production and living space more than the macro factors. By contrast, the macro factors influenced the evolution of ecological space more than the micro factors. For living space evolution, the most important factors were the city resident (0.215), the entrepreneur (0.127) and the environmentalist (0.123). This indicates that the decision-making behaviors of city residents play the most important role in urban living space development. For production space evolution, the most important factors were the rural resident (0.202), the entrepreneur (0.147), and the city resident (0.098). This means that production space development depends more on human wishes than on natural resources and conditions or the development of science and technology. For ecological space evolution, the most important factors were the location condition (0.230), natural condition (0.150), and neighborhood influence (0.142). This indicates that the location and natural condition have an important influence on the development of ecological space, and with the increase in awareness of environmental protection, human intervention in the evolution of ecological space is decreasing. It has instead developed according to local conditions in Xuzhou in the past decades.

## 5. Discussions

The scientific planning of urban space is an important way to realize urban sustainable development. The precise simulation and understanding of the leading factors in urban space evolution are essential prerequisites for urban space planning. However, there are still some defects in existing models. For example, the econometric statistics model and the system dynamics model have a strong ability to predict the size of urban space evolution, but they are unable to simulate the variation in urban spatial distribution [36]. For another example, the cellular automata model is a key milestone in the development of urban spatial simulation technology, which can simulate not only the variation in urban space size but also the variation in urban spatial distribution, but it is unable to simulate the effects of macro-scale factors (e.g., policy, economy, culture) on urban space evolution, and nor can it simulate the decision-making behaviors of different urban agents (e.g., resident, entrepreneur, environmentalist) [37]. With the development of artificial intelligence (AI) technology, the study of a joint model of MAS and CA has become a hot spot for urban space evolution simulation. This type of model can simulate the influences of macro factors (e.g., natural environment, geographical location, policy) and micro factors (e.g., different urban agents) on urban space evolution [38]. Therefore, the joint model of MAS and CA has a stronger ability to simulate urban space than other models.

However, the current CA + MAS model does not consider the correlations between macro and micro factors. It is assumed that the effects of macro and micro factors are completely independent, which is inconsistent with the facts, as macro factors (e.g., the natural condition, government policy) often restrict the decision-making behaviors of micro factors (e.g., residents, entrepreneurs, environmentalists). Meanwhile, the preferences of micro factors also have an important influence on the decision-making behaviors of macro factors. Therefore, if the interaction between and mutual influence of macro and micro factors are neglected, the simulation precision of CA + MAS model will decrease. In addition, the simulation objects of current models are usually unit plots with different types of land use (e.g., woodland, grassland, water body). Therefore, the simulation result ascribes a specific type of land use to each unit plot. However, the type of land use may be different from the urban planning for a given unit plot (e.g., traffic planning, garden planning, land planning) [39]. Therefore, if multiple plans are implemented in the same place at the same time, it will lead to difficulties in decision-making and implementation (e.g., the conflict areas) [40].

To alleviate the above problems, a macro–micro joint decision model is proposed based on the CA + MAS model in this study to improve the simulation of urban space evolution. The simulation objects are unified into production, living and ecological space for the convenience of unified implementation of multiple plans. For validation of the proposed model, remote sensing images, geographic information and socio-economic data from Xuzhou, China between 2000 and 2020 were collected and tested. The results proved that the simulation and prediction precisions of the proposed model were better than those of current models (e.g., CA, CA + MAS) for urban space evolution simulation, particularly for the simulation of living space evolution.

It is very important for urban planning and sustainable development strategies that the precision of PLE evolution simulation models be improved, because urban space evolution simulation models are often used to compare the implementation effects of different urban planning schemes. Therefore, it is beneficial to select the optimal planning scheme for realizing sustainable urban development, if the model simulation precision is high. Otherwise, this can result in faulty government decision-making and the waste of urban resources if the model simulation precision is poor. A high-precision simulation model helps urban planners discover the problems associated with different plans in advance, and is also an important tool to analyze the effects of different factors on the evolution of urban space. Therefore, it is very useful to grasp the rules governing urban space evolution and operation mechanisms.

However, there are still some limitations of the proposed model in this study. (i) The urban space is expressed as a regular grid (30 m × 30 m) in the proposed model, but actual urban space is an irregular polygon. This led to inconsistencies between the simulation result and the RS imaging-observed results, as well as a decrease in simulation precision [41]. (ii) In theory, the smaller the area of unit space, the higher the precision of urban space simulation. However, the computational burden increases at exponential levels with decreasing areas of unit space. Therefore, the selection of optimal geographic unit scale for simulation of urban space evolution remains an unsolved problem. For larger areas (e.g., an urban agglomeration or economic zone), an adaptive theory should be applied to define the grid scale. Regions with rapid land use change could be defined using a small grid (e.g., 30 m × 30 m), while regions with slow land use change could be simulated using a large grid (e.g., 300 m × 300 m). On the one hand, the differentiating grids could improve simulation accuracy; on the other hand, they could ensure operational efficiency. (iii) The estimated precision of the factor initial weight is strongly related to the sample selection when using a statistical method. Therefore, the selection of representative and diverse samples is a key problem. In this study, an even sampling strategy was adopted to select the experimental samples. However, this was not an optimal solution. In addition, the variation in urban population size and spatial distribution is an important factor that affects urban space evolution [42]. In this study, the information on urban population size was used to predict the variation in living space size, but the data of population spatial distribution is not used. Therefore, urban space evolution simulation models should be further investigated considering the variations in population spatial distribution in the future.

## 6. Conclusions

The accurate simulation of urban space evolution and understanding of the leading factors are key issues to improve the sustainability of urban development. In this study, a macro–micro joint decision model was constructed based on the CA + MAS model in order to improve the ability to simulate urban space. A method of factor weight determination was proposed to analyze the effects of different factors on urban space evolution. For validation of the proposed model and method, experimental data (e.g., RS data, GIS data and socio-economic data) were collected and tested. The results proved that the proposed model and method were valid and reliable, and could improve the simulation and prediction of urban space evolution. The main conclusions of this study are as follows:

(1) The simulation precision of urban space evolution from the CA + MAS model was better than that from the CA model, because the decision-making behaviors of different urban agents (e.g., resident, entrepreneur, environmentalist) were considered in the CA + MAS model and not the CA model. Moreover, if the interactions and influences of macro and micro factors were considered (e.g., CA + MAS + Correlation model), the simulation precision of the CA + MAS model could be further improved. In this study, the simulation precisions (Kappa coefficient) of urban space evolution from the CA, CA + MAS and CA + MAS + Correlation models were 87.14%, 89.63% and 92.31%, respectively. The improvement in living space simulation precision was the most significant when using the CA + MAS+ Correlation model, compared with simulation of other types of spaces.

(2) The prediction precisions of CA, CA + MAS and CA + MAS + Correlation model were worse than their simulation precisions, as the factor weights in prediction models were replaced with those from the simulation models, disregarding changes that occurred during the prediction period. Therefore, errors were inevitably introduced. However, the prediction precision of the CA + MAS + Correlation model remained better than that of the CA and CA + MAS models, and the improvement in prediction precision was larger than that in simulation precision using the CA + MAS + Correlation model, compared to the CA and CA + MAS models. It was proved that the CA + MAS + Correlation model had a stronger ability to predict PLE space evolution than the CA and CA + MAS models.

(3) According to the results of factor weight determination, it was determined that the effects of micro factors on the evolution of living and production space were greater than those of macro factors. This indicated that the influences of desires and behaviors of human beings on the evolution of living and production space are increasing, correlating with the development of science and technology. The decision-making behaviors of city residents played the most important role in urban living space development. In the evolution of production space, rural residents and entrepreneurs had more influence than the other factors. By comparison, the effects of macro factors on the evolution of ecological space were more significant than those of micro factors, where the three most important factors were the location condition, neighborhood influence and natural condition. This means that the location and natural condition have more influence on the evolution of ecological space than the other factors.

According to the above analysis and conclusions, some policy implications are proposed to improve the sustainability of urban development. At present, the micro factors (e.g., city and rural residents and entrepreneurs) are the leading factors in the evolution of living and production space. Therefore, active policies should be formulated to strengthen the ideological guidance for these micro individuals, help them establish scientific views of urban space utilization, and realize the ordered development of living and production space. In addition, macro factors (e.g., location condition, natural environment) have the most important influence on the evolution of ecological space. Therefore, urban ecological space planning should closely link with the local environmental and natural conditions, to improve urban ecological carrying capacity and realize urban sustainable development.

## Figures and Tables

**Table 1:** Performance comparison of current models for urban spatial evolution simulation.

Performance	ES ^(a)^	SD ^(b)^	CA ^(c)^	MAS ^(d)^
Scale changes simulation	Strong	Strong	Strong	Strong
Spatial distribution simulation	Weak	Weak	Strong	Strong
Time varying simulation	Weak	Normal	Strong	Strong
Macro factors simulation	Strong	Strong	Weak	Strong
Micro factors simulation	Weak	Weak	Normal	Strong
Model operation mechanism	Top-down		Bottom-up	

^(a)^ econometric statistics model; ^(b)^ system dynamics model; ^(c)^ the cellular automata model; ^(d)^ the multi-agent system model.

**Table 2:** Study data sources and contents.

Data Type	Content	Time	Source
RS image data	GF ^(a)^-2 1m × 1m RS ^(b)^ image	2000–2020	Natural Resources Satellite RS Cloud Service Platform
Urban GIS ^(c)^ data	Vector files of administrative division Road	2018	Geographical Information Monitoring Cloud
Socioeconomic statistics	Population, industrial economy, natural resources	2000–2020	Literature, statistical yearbooks

^(a)^ GaoFen-2 remote sensing images; ^(b)^ Remote sensing; ^(c)^ Geographic information system.

**Table 3:** Statistical precisions of PLES evolution simulation from three schemes Unit: %.

Evaluated Object	Scheme 1	Scheme 2	Scheme 3
Model	CA	CA + MAS	CA + MAS + Correlation
Kappa	87.14	89.63	92.31

**Table 4:** Statistical precisions of PLES evolution prediction from three schemes Unit: %.

Evaluated Object	Scheme 1	Scheme 2	Scheme 3
Model	CA	CA + MAS	CA + MAS + Correlation
Kappa	84.59	86.19	89.33

**Table 5:** Initial weight determination results of factors in living space evolution simulation.

Elevation	Slope	Distance to Water	Distance to Road	Distance to Center	Government Planning	Type of Land	Neighborhood Influence
0.028	0.104	0.035	0.064	0.043	0.076	0.047	0.056
Influence of Micro factor	Protected land	House Price	Hospital	Mall	School	Distance to Ecological Space	Influence of Macro factor
0.014	0.076	0.095	0.109	0.058	0.089	0.064	0.042

**Table 6:** Initial weight determination results of factors in production space evolution simulation.

Elevation	Slope	Soil Quality	Distance to Water	Distance to Road	Distance to Center	Type of Land
0.042	0.046	0.052	0.059	0.106	0.043	0.109
Neighborhood Influence	Influence of Micro factor	Protected land	Land Price	Distance to living space	Distance to Ecological Space	Influence of Macro factor
0.065	0.032	0.184	0.091	0.102	0.035	0.034

**Table 7:** Initial weight determination results of factors in ecological space evolution simulation.

Elevation	Slope	Distance to Water	Distance to Woodland	Distance to Grassland	Type of Land	Neighborhood Influence
0.069	0.076	0.072	0.148	0.076	0.064	0.091
Influence of Micro factor	Protected land	Distance to production space	Distance to living space	Distance to Ecological Space	Influence of Macro factor	/
0.081	0.037	0.056	0.057	0.149	0.024	/
